# Feasibility of multimodal 3D neuroimaging to guide implantation of intracranial EEG electrodes

**DOI:** 10.1016/j.eplepsyres.2013.08.002

**Published:** 2013-11

**Authors:** Roman Rodionov, Christian Vollmar, Mark Nowell, Anna Miserocchi, Tim Wehner, Caroline Micallef, Gergely Zombori, Sebastien Ourselin, Beate Diehl, Andrew W. McEvoy, John S. Duncan

**Affiliations:** a Department of Clinical and Experimental Epilepsy, UCL Institute of Neurology, London, UK; b Epilepsy Society, MRI Unit, Chalfont St Peter, UK; c Epilepsy Center, Department of Neurology, University of Munich, 81377 Munich, Germany; d Department of Neurosurgery, National Hospital for Neurology and Neurosurgery, London, UK; e Department of Clinical Neurophysiology, National Hospital for Neurology and Neurosurgery, London, UK; f Department of Neuroradiology and Neurophysics, National Hospital for Neurology and Neurosurgery, London, UK; g Centre for Medical Image Computing, UCL, London, UK

**Keywords:** Multimodal, Neuroimaging, Epilepsy, Surgery

## Abstract

**Background:**

Since intracranial electrode implantation has limited spatial sampling and carries significant risk, placement has to be effective and efficient. Structural and functional imaging of several different modalities contributes to localising the seizure onset zone (SoZ) and eloquent cortex. There is a need to summarise and present this information throughout the pre/intra/post-surgical course.

**Methods:**

We developed and implemented a multimodal 3D neuroimaging (M3N) pipeline to guide implantation of intracranial EEG (icEEG) electrodes. We report the implementation of the pipeline for operative planning and a description of its use in clinical decision-making.

**Results:**

The results of intraoperative application of the M3N pipeline demonstrated clinical benefits in all 15 implantation surgeries assessed. The M3N software was used to simulate placement of intracranial electrodes in 2 cases. The key benefits of using the M3N pipeline are illustrated in 3 representative case reports.

**Conclusion:**

We have demonstrated feasibility of the developed intraoperative M3N pipeline which serves as a prototype for clinical implementation. Further validity studies with larger sample groups are required to determine the utility of M3N in routine surgical practice

## Highlights

• Developed pipeline for multimodal image integration to guide epilepsy surgery.
• Reported the key principles of the pipeline and its detailed description.
• Demonstrated feasibility of the developed pipeline for multimodal image integration.
• Illustrated potential benefits of intraoperative use of the pipeline.

## Introduction

Multimodal 3D neuroimaging (M3N) applied to navigation during neurosurgical operations is a technique which utilises pre- and intraoperative display of multimodal data in the neuronavigation system. The benefits of M3N have been recognised during resection of brain tumours (González-Darder et al., 2010; Nimsky et al., 2011; Spena et al., 2010; Tharin and Golby, 2007). There are few reports of intraoperative use of M3N for surgical treatment of refractory focal epilepsy (Holowka et al., 2004; Murphy et al., 2004; Wellmer et al., 2010).

The area of brain that needs to be removed to achieve seizure freedom, often referred to as the epileptogenic zone (EZ), may not be associated with a clear structural abnormality and may impinge on eloquent cortex, damage to which would cause a new deficit (Duncan, 2010). In these cases, it may be necessary to place subdural and/or intracerebral depth electrodes to define the site of seizure onset (SoZ), that must be removed to give the optimal chance of seizure freedom, and its relation to eloquent cortex (Duncan, 2011). Spatial sampling from intracranial electrodes is limited and placement carries significant risk (Tanriverdi et al., 2009). Therefore, electrode placements need to be of optimal effectiveness and efficiency.

The visualisation of multimodality neuroimaging data in 3 dimensions offers increased sophistication and accuracy for electrode placement. Structural and functional imaging of several different modalities allows inferences to be drawn on the location of the SoZ, and also eloquent areas and critical cerebral structures. This data may include structural MRI, tractography, vascular imaging, functional MRI of eloquent functions and epileptic activity, cerebral perfusion (SPECT – Single-Photon Emission Computed Tomography), glucose metabolism (FDG-PET – Fluorodeoxy-d-Glucose Positron Tomography), magnetic source imaging (MSI) and electrical source imaging (ESI). Traditionally, such datasets have been viewed individually, in 2-dimensional slices. Advances in visualisation software and computing hardware have led to the potential ability to place diverse datasets in the same individual patient brain space and to view such data in 2- and 3-dimensions, with interactive visualisation of the brain surface and deep structures.

We report the feasibility of intraoperative use of M3N to guide implantation of icEEG electrodes, describe the methodology with our instrumental platform and illustrate the implementation of the platform in 15 individuals with drug-resistant focal epilepsies.

## Methods. Implementation of M3N for operative planning

The M3N pipeline is used to support clinical decision making at all stages including the discussion of treatment strategy, planning and performing icEEG electrode implantation, icEEG analysis, resective surgery and analysis of postoperative outcome. This pipeline would be widely applicable across sites, and can import data from a wide range of imaging protocols.

**Fig. 1:**
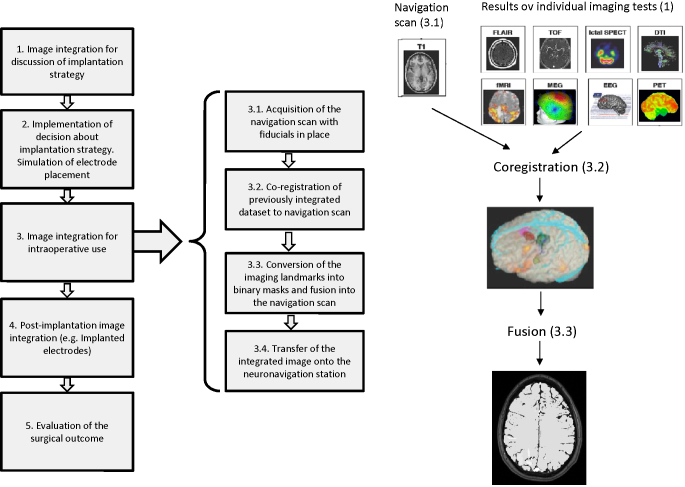
Workflow of M3N for presurgical evaluation of epilepsy patients with focus on the details of the data integration stage for guidance of implantation.

The detailed pipeline of M3N is described in Supplement 2 and illustrated in Fig. 1. The typical parameters (matrix size and voxel size) of the images utilised in our implementation of M3N are listed in the Table 1. These are useful for approximate estimation of the required computer memory and processing power.

The M3N pipeline requires image integration software. The key requirements to this software are: (a) variety of image processing tools to support typical tasks outlined in this section and Supplement 2; (b) intuitive and efficient way of displaying and manipulating 3D imaging information; (c) ability to rapidly combine basic image processing tools into workflows for easy and quick application. Amira 5 software (Visualization Sciences Group, USA; www.vsg3d.com) fulfils these criteria and was used to carry out all image integration processing.

Intraoperative use of M3N begins with previously fused multimodal image data (see stage 3 on the left hand side diagram on the Fig. 1). This data includes some of the landmarks useful for identification of SoZ: abnormal tissue as segmented from diagnostic T1 and T2 MR images; cortex generating interictal epileptic activity as indicated on 3D mapping of MEG, EEG and EEG-fMRI data; cortex showing increased metabolism during seizures as shown by SPECT. In addition it is crucial to visualise and present critical structures such as cortico-spinal tract and optic radiation that are demonstrated using DTI tractography, arteries and veins and eloquent cortex (e.g. motor and language functions localised using fMRI).

**Fig. 2:**
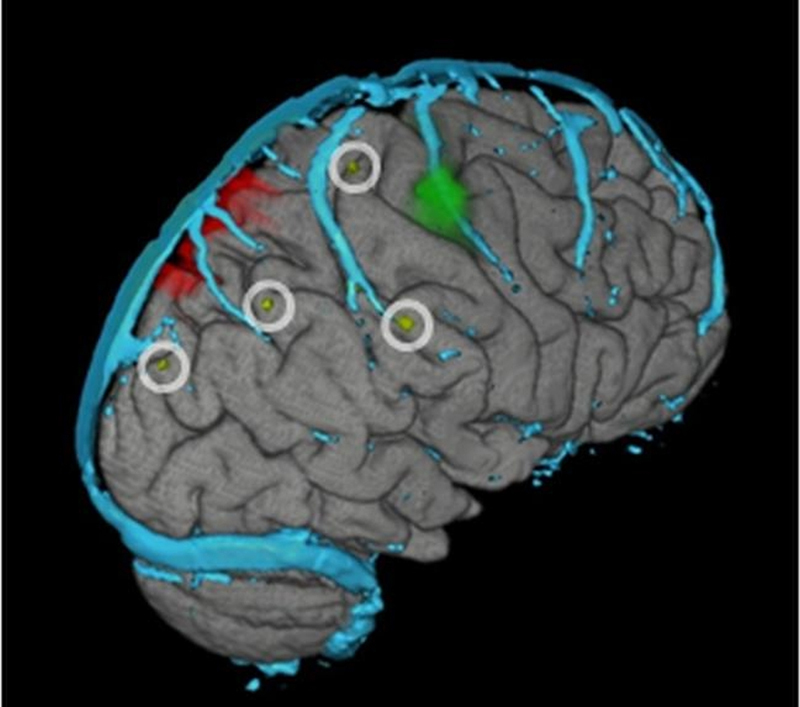
3D models simulating intracranial depth electrodes. Patient 13. Depth electrode entry points chosen at the crown of gyri and avoiding pial boundaries. Entry points of the 3D models of the depth electrodes are seen in the middle of the white circles. Red – lesion; green cluster – hand motor area labelled using fMRI; cyan – cortical veins. (For interpretation of the references to color in this figure legend, the reader is referred to the web version of this article.)

**Fig. 3:**
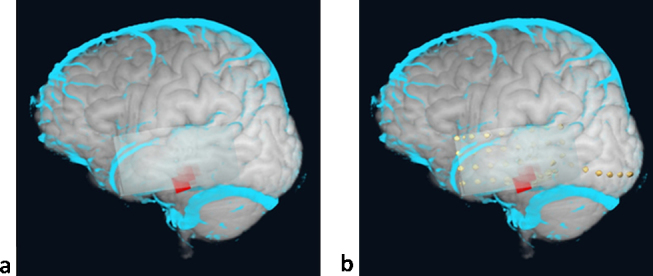
3D model simulating intracranial grid electrode. Patient 15. 3D model of the grid electrode (the transparent square surface over Lt temporal lobe) before (a) and after (b) placement of the intracranial electrodes – individual yellow contacts on (c). Red – lesion; cyan – cortical veins. (For interpretation of the references to color in this figure legend, the reader is referred to the web version of this article.)

**Fig. 4:**
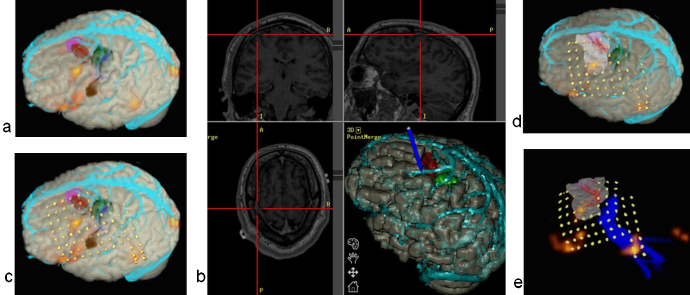
Use of M3N during implantation surgery of Patient 2 (left lateral 3D views). (a) pre-implantation; (b) during implantation. The dark blue pointer on (b) shows Stealth navigation probe when it was placed on the large cortical vein; (c) post-implantation; (d,e) post-resection with highlighted removed cortex. Colour codes: red – lesion; green – hand motor areas mapped using fMRI; magenta – ictal hyperperfusion from SPECT; orange – expressive language areas mapped using fMRI; brown (on a,c; lt precentral gyrus) – spike-related increase of BOLD signal mapped using EEG-fMRI; cyan – surface veins; blue (e) – corticospinal tract. (For interpretation of the references to color in this figure legend, the reader is referred to the web version of this article.)

**Fig. 5:**
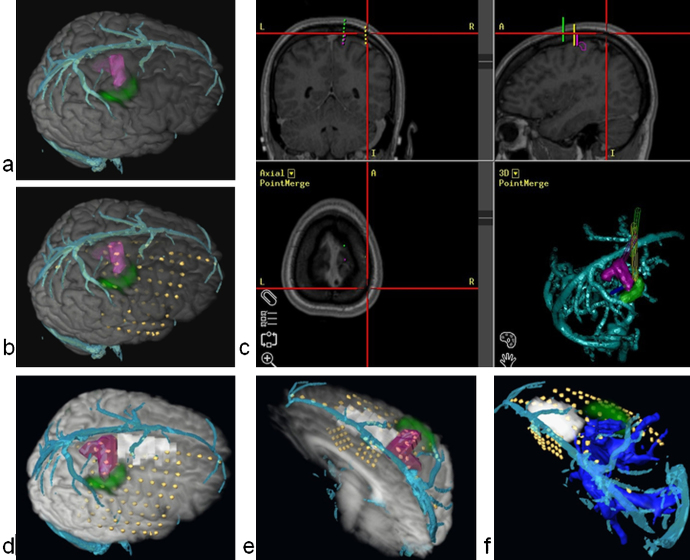
Use of M3N during implantation surgery of Patient 5. (a) pre-implantation; (b) post-implantation; (c) during implantation. The dark blue pointer on (c) shows Stealth navigation probe when it was placed on the surface vein; (d,e,f) post-resection with highlighted removed cortex. Colour codes: green – hand motor area mapped using fMRI; magenta – area of hypometabolism according to ictal SPECT; cyan – surface veins; dark blue (f) – cortico-spinal tract. (For interpretation of the references to color in this figure legend, the reader is referred to the web version of this article.)

**Fig. 6:**
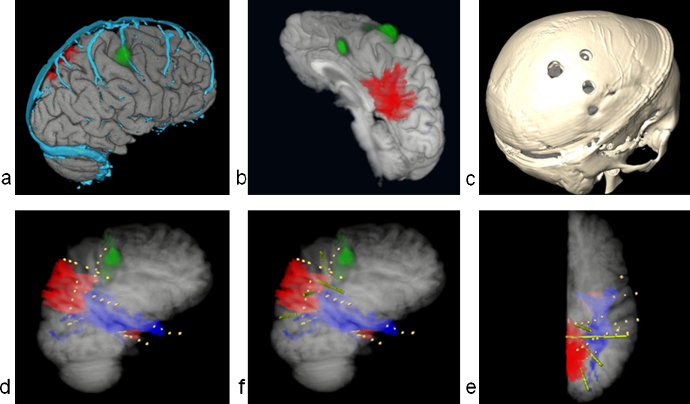
Use of M3N for implantation of depth electrodes in Patient 13. (a,b) pre-implantation; (c,d,e,f) post-implantation. Only right hemisphere is shown. Colour codes: red – lesion; light green – preoperatively simulated electrodes; dark green – hand motor area mapped using fMRI; cyan – surface veins; dark blue – optic radiation; yellow – individual contacts of implanted electrodes on d,e,f. (a,d) – right lateral 3D views; (b) – left posterior-lateral view; (e) – medial view; (f) – posterior superior view of the right hemisphere. (c) – skull showing holes used to perform the implantation (segmented from CT image). (e,f) – electrodes modelled preoperatively using M3N (Amira) compared with placed electrodes. (For interpretation of the references to color in this figure legend, the reader is referred to the web version of this article.)

The algorithm for the intraoperative use of M3N comprises the following steps (see stages 3.1–3.4 on Fig. 1):

• The anatomical MR image (T1-weighted with gadolinium contrast) with skin fiducials on the scalp and used for navigation (navMRI) is obtained immediately prior to surgery. The fiducials used for coregistration of the image with the patient's head are clearly visible on the image. This image represents the basis to which binary spatial labels representing all surgically relevant 3D models are fused. This image is not spatially transformed during further processing, to facilitate seemless coregistration of the images that contain all 3D models with the navMRI on the neuronavigation system.
• The anatomical T1-weighted image obtained previously and which defines the spatial basis for preoperative image fusion is coregistered with the navMRI dataset using a rigid body transformation (rotation around and translation along three axes). The resulting transformation is applied to all other imaging modalities, providing useful landmarks for inferring the spatial characterisation of the SoZ and consequently useful intraoperatively.
• The images of landmarks are converted into a set of homogenous masks for fusion with the navMR image. Consecutive masks are assigned different intensities to facilitate differentiation between them in the resulting image.
• Planning depth electrode implantation. The placement of depth electrodes can be simulated by adding 3D models representing the size and location of both depth and subdural electrodes (Figs. 2 and 3). The display of depth electrodes facilitates the avoidance of pial boundaries and major blood vessels, and allows for targeting of structural and functional data.
• The multimodal masks are fused into the navMRI image which becomes the image-container of the 3D models (Figs. 4–6). Note, in cases when it is important to appreciate two or more overlapping areas, the area of overlap can be encoded as an extra mask. The intensity encoding this additional mask has to be between the intensities encoding the two overlapping areas. This approach allows complete representation of overlapping areas.

The resulting 3D image, the image-container of landmarks is exported in DICOM format, recorded on a CD and transferred onto the Stealth Station (MedTronic Inc). The image-container is co-registered with the navigation T1 MR image and the 3D models are re-created using neuronavigation software.

The image-container carries out only one function–transport of the 3D models into the neuronavigation system in the correct anatomical space. The diagnostic navigation images required during the surgery are uploaded into the neuronavigation station following procedures recommended by the manufacturer. The time consuming process of preparing the image-container is a necessary step as the neuronavigation systems use internal data formats to store complex datasets including 3D models and implantation plans. The advantage of this approach is that the resulting image-container can be utilised by any neuronavigation system which exports DICOM images and provides tools for manual segmentation of 3D labels into 3D surface models.

Among the benefits of the pipeline is ability to apply it to any data set. This flexibility means that the pipeline is not limited to the modalities reported in the Results section, but can incorporate new imaging data with clinical potential.

Amira software is not CE marked or FDA approved for clinical use. In order to avoid registration errors, the results of coregistration were reviewed and confirmed as adequate to the surgical procedure by at least two experts in neuroimaging.

All visualisations, including the tool OrthoView showing orthogonal projections, in Amira are implemented in 3D space. This allows unequivocal identification of right/left. Our experience shows that Amira is consistently correct in visualising DICOM and Nifti images (tested on over 400 images from seven MR, PET/SPECT, CT scanners). However, the user should be cautious when interpreting laterality of any images imported in Analyze format as these are less reliable in identification of laterality. The tools supporting 2D image viewing in the form of 3 projections are also available as secondary option.

The benefits of intraoperative use of M3N were reported by operating neurosurgeons by comparing clinical decision-making before and after the display of M3N results, and by summarising use at the end of each case. However, the focus of this report is to describe the feasibility of implementing the intraoperative M3N pipeline.

## Results

The intraoperative M3N pipeline was implemented for use during implantation of icEEG electrodes at the National Hospital for Neurology and Neurosurgery (Queen Square, London, UK). M3N was available on the neuronavigation system for 15 consecutive implantation surgeries (Supplement 1). The simulation of placement of the depth electrodes was performed in the two most recent cases.

The research protocol for this study was approved (registration number 12/LO/0377) by Ethics Committee of the NHNN. Informed consent was obtained to acquire the imaging data. The benefits from the intraoperative use of M3N were noted by the two operating neurosurgeons (AWM, AM) for every surgery (Supplementary material 1).

The following three representative case reports illustrate the benefits of utilisation of M3N including a case in which the depth electrodes were pre-operatively simulated as 3D models and these data were used intraoperatively.

Case 2 *grid and depth electrodes.*

A 30-year old man presented with refractory left frontal lobe epilepsy from the age of 12 years, with most frequent seizures characterised by head and eyes version to the right and speech arrest. MRI showed a small area of focal cortical dysplasia (FCD) in the bottom of a sulcus within the left superior frontal gyrus. SPECT showed increased ictal blood flow around the area of FCD. Motor hand and language fMRI showed the proximity of these eloquent functions. Tractography demonstrated the corticospinal tract (CST). MR venography demonstrated the cortical surface veins and their relation to the structural lesion, gyral anatomy and eloquent cortex (Supplement 1, Fig. 4). The models were useful for planning the craniotomy and for identifying the central sulcus and the lesion. The set of 3D models was available intraoperatively on the neuronavigation system during the implantation. The operating neurosurgeon confirmed anatomical accuracy of the models using cortical veins as landmarks (Fig. 4b). The 3D model of cortical veins offered a guide to brain shift and thus facilitated gross correction when placing depth electrodes.

The seizure onset zone identified with intracranial EEG, areas of cortex involved in rapid propagation of ictal activity and the dysplastic lesion were removed (see post-resection image fusion on Fig. 4d,e) preserving CST and hand motor area. The patient had, as predicted, a minor expressive speech deficit which improved within 3 months. The patient has remained seizure free 15 months following surgery.

M3N led to accurate placement of intracranial electrodes for the purposes of recording seizures and mapping eloquent function and was helpful for guiding lesional resection in a highly eloquent area.

Case 5 *grid electrodes.*

A 28-year old woman presented with refractory frontal lobe epilepsy diagnosed at age 10 years. The seizure semiology characterised by tonic and bilateral asymmetric tonic seizures suggested right frontal lobe onset with involvement of the supplementary motor area (SMA). No abnormalities were found on structural MRI. Ictal SPECT showed hyperperfusion in the right superior frontal gyrus, in the vicinity of the SMA (Fig. 5) (Supplement 1).

The 3D models showing major cortical veins, area of ictal hyperperfusion, left hand motor area were available on the neuronavigation system during implantation surgery. The location of the ictal hyperperfusion and the 3D model of cortical veins were used as landmarks to place small 4 × 4 grid electrodes on the medial aspect of the superior frontal gyrus (Fig. 4e) and to locate the central sulcus. The brain shift was estimated as relatively minor given the size of the craniotomy.

The SoZ was confirmed by the icEEG and was removed (Fig. 5d,e,f) preserving the CST (Fig. 5f) and motor cortex. As expected following removal of the SMA, there was mild left hemiparesis affecting mainly the left upper extremity for 2 weeks which then resolved completely. Histological analysis showed FCD type IIb. The patient discontinued all medication 4 months post-operatively. Twelve months following the surgery the patient remains seizure free.

M3N demonstrated concordance between ictal hyperperfusion and icEEG findings in a case of non-lesional epilepsy and guided the resection. The visualisation of the left hand motor area and CST allowed this resection to be performed safely.

Case 13 *depth electrodes.*

A 42-year old woman presented with refractory epilepsy. She initially developed epilepsy aged 9 years, and underwent a right anterior temporal lobe resection aged 11. This resulted in a change of seizure semiology, with the new seizures characterised by a left leg somatosensory aura, followed by left arm tonic posturing and secondary generalisation. The implantation strategy (Supplement 1) was based on seizure semiology, scalp EEG and the ischaemic lesion visible on MRI in the precuneus and medial posterior temporal lobe. fMRI demonstrated primary sensory and motor cortex and tractography defined the optic radiation (Fig. 6).

The location of the depth electrodes was planned preoperatively using the M3N pipeline, enabling precise electrode positioning to define the seizure onset zone and primary somatosensory cortex, and to minimise the risk of damaging large surface cortical veins (Fig. 6). Post implantation imaging showed that the preoperative electrode placement using the M3N pipeline was accurately used in practice.

The plan and3D models made preoperatively, were uploaded to the neuronavigation system, and used in the operating room to inform the insertion of the intracranial electrodes.

The seizure onset zone was defined in the posterior medial right temporal lobe and resection has been scheduled.

Automated cortex segmentation was complicated in the area of previous temporal lobe resection which resulted in masking out small amount of tissue (Fig. 6 d,f). For this reason the cortical anatomy was estimated using the navigation T1 image.

M3N was useful for demonstrating the proximity of the lesion to eloquent brain, and in creating the planned trajectories for electrode implantation with avoidance of vascular injury.

## Discussion

The application of the intraoperative M3N pipeline has shown that this approach is feasible and has potential utility for guiding the insertion of intracranial EEG electrodes and the resection of an epileptic focus. Our experience demonstrates that the M3N pipeline can be implemented into standard clinical practice in a busy epilepsy surgery centre.

We are not aware of any reports on the systematic use of M3N for all epilepsy surgery implantation cases, incorporating PET, SPECT, MEG, fMRI and tractography.

There are a few reports of application of intraoperative multimodal imaging aiming to guide electrode implantation (Cohen et al., 2010; Daga et al., 2012; Stadie et al., 2008; Wellmer et al., 2010). However, present experience is limited to evaluation of effect of preoperative (Cohen et al., 2010; Stadie et al., 2008) or intraoperative (Daga et al., 2012; Wellmer et al., 2010) use of individual modalities. In general, these reports fall into two groups: techniques to improve the yield of identifying the epileptogenic zone, and techniques to identify regions of interest to avoid surgical complications.

The use of functional imaging, including FDG-PET, T2 Fluid Attenuated Inversion Recovery (FLAIR) MRI, ictal SPECT, has been reported in difficult, non lesional cases to identify previously occult pathology (Rastogi et al., 2008). The export of these data to neuronavigation systems places it in an anatomical framework, and allows the surgeon to accurately locate these regions of interest. A recent report describes a novel MRI processing technique, which can identify areas of FCD (Wellmer et al., 2010). These data can be transferred to neuronavigation systems, for use in theatre. There are reports of the use of tractography to guide anterior temporal lobe resections, avoiding damage to Meyer's loop (Daga et al., 2012). The integration of vascular imaging with structural imaging to guide electrode placement is a well established technique, first described by Talairach and employed in stereotactic EEG for over 30 years (Talairach and Szikla, 1980).

The key feature of the intraoperative M3N pipeline is the routine display of all available and relevant structural and functional neuroimaging data to aid informed decision-making with necessary precision. The particularly useful aspects are:

• The coregistration and simultaneous display of multiple datasets, including T1, T2-weighted and FLAIR MRI acquisitions with lesions highlighted.
• The inclusion of derived structural data, particularly critical white matter tracts, such as cortico-spinal tract and optic radiation, defined with probabilistic tractography.
• Delineation of major cortical veins, obtained with MR venography.
• The display of eloquent motor, sensory, language and visual cortex, identified with fMRI.
• The addition of other key functional data that infer the location of the epileptic focus: hypometabolism identified with FDG-PET, ictal hyperperfusion identified with SPECT; fMRI of interictal epileptic activity obtained with EEG-f MRI; electrical and magnetic source imaging.
• The simulation of depth and subdural electrodes as 3D objects.
• The utilisation of the standardised colour scheme for display of 3D models.

The following features specific to planning implantation contribute to useful functionality of our pipeline:

1. The simulation of depth electrodes as 3D objects allows optimal selection of target sites and visualisation of the intracerebral course of the electrode. This is done outside the neuronavigation system, allowing greater access and close consultation with neurologists and neurophysiologists. The 3D models of the intracranial electrodes may then be uploaded on the Stealth workstation to guide the actual electrode insertion.
2. Subdural grid electrodes may be simulated (Fig. 3b,c) and placed on the rendered surface of the brain, facilitating the placement of the subdural grid, to minimise edges of the grid overlaying major surface veins, and to ensure good coverage of the presumed epileptic focus and nearby eloquent cortex, so that functional mapping may be carried out effectively. Additionally, this is valuable for assisting the planning of the craniotomy.

A further potential benefit of the pipeline, noted during the course of its implementation in clinical practice, is its enhancement of communication and decision-making between different specialists (neurologists, neurophysiologists, neurosurgeons and neuroradiologists) involved in patient care.

Current software for M3N applied to neurosurgery fall into three categories:

1. Basic planning on commercially available neuronavigation systems (Medtronic Stealth Cranial, Medtronic Inc, USA; BrainLab Cranial Navigation, Brainlab AG, Germany);
2. Specialised planning software as adjunct to neuronavigation software (StealthViz, Medtronic Inc; iPlan, Brainlab AG)
3. Stand-alone specialised planning software packages (BioImageSuite, Yale University, USA; 3D Slicer, Brigham and Women Hospital, USA; Analyze, Mayo Clinic, USA).

Commercially available neuronavigation systems allow the import of dedicated navigation data sets from MRI and CT, followed by automated coregistration. Thus it is possible to merge T1 and T2 weighed MRI with CT. It is not possible to import other structural or functional data. There is a basic functionality for building simple 3D model sets according to thresholding, but there is very limited functionality with regards to data processing and analysis, visualisation and presentation.

Commercially available planning software packages, such as StealthViz, have advanced functionality, with coregistration of a wide range of data sets and mature segmentation tools. However, it is not possible to perform surgical planning with his software.

The most versatile systems are the stand-alone specialised multifunctional software packages (e.g. BioImageSuite, 3D Slicer, Analyze) that are developed outside of industry. These are used in a range of disciplines in basic and clinical science. The major advantage of these packages is that they are not dedicated to solving specific well-defined problems. This ‘non-specialisation’ means that there is added flexibility in the processing, manipulation and display of data sets, and an added range of tools to choose from.

Our choice of software platform for M3N was Amira. This software combines flexibility, simplicity of use, variety of functions and interactive image display, and allowed the simulation of implanted hardware for epilepsy surgery planning. The flexibility of Amira can be illustrated by its ability to implement simulation of electrode placement. In this way Amira-based instrumental part of M3N supports all image processing tasks arising in the process of surgical treatment of epilepsy.

We anticipate three barriers to the widespread adoption of M3N in clinical practice:

• organisational infrastructure;
• accuracy;
• validity.

Firstly, M3N is a complex addition to a clinical pathway, which requires considerable changes in organisational infrastructure. The first requirement is the availability of the range of imaging modalities, along with the time and expertise to perform relevant pre-processing of data. The second requirement is the availability of expert data integration and validation, which requires specialist training and can be costly in terms of man hours. Currently, a complete data set for a patient requires 8–12 hours work with the use of at least 4 GB of RAM. The third requirement is a common consensus by the entire multi-disciplinary team to engage with the programme, accepting that adoption is accompanied by a learning curve in how best to present and use the data. Once the Amira-based instrumental part of M3N is implemented into the clinical context of an epilepsy centre it can be successfully operated either by a radiology technician or a clinical scientist.

Secondly, the accuracy of M3N is complex and varied. The old adage by Aristotle, “You get out what you put in” is especially pertinent to M3N, where some data sets will be reliable and reproducible such as structural MRI, and other sets will have considerable inter-user variability such as tractography (Heiervang et al., 2006). Some data sets are further limited by their own biophysical principles; for example, blood oxygen level dependent signal in fMRI is an indirect measure of neuronal activity, and tractography is a presumption of white matter connectivity. Interpretation of these within an integrated data set requires differential levels of caution and confidence.

Integration and presentation of multimodality imaging is a step-wise process of spatial coregistration, and each step carries a margin of error. For this reason it is essential that coregistration is checked manually, and that errors in anatomical localisation such as laterality, are checked at each stage. We aim for a coregistration accuracy of 2–5 mm, where lower accuracy is similar to the spatial resolution of fMRI, tractography, PET and SPECT. Targeting deep central structures requires anatomical precision of 1–2 mm, and for this high resolution anatomical MRI is used. We did not aim to perform quantitative analysis of coregistration accuracy provided by Amira and the result of every coregistration was evaluated visually.

For M3N to be available to the neurosurgeon in theatre, the data sets are exported to a neuronavigation system, and a further registration takes place between the 3D model and the patients head. This adds a further margin of error, which is dictated by the neuronavigation software and quality of registration. The accuracy of this spatial registration further deteriorates as the surgical procedure takes place, due to brain shift. This is the intraoperative displacement and distortion of the brain that inevitably occurs during operations, as a result of CSF loss, gravity, brain swelling and brain resection. The aggregation of these margins of error has to be considered as the neurologist or neurosurgeon integrates this additional tool into clinical practice.

Finally, a further barrier to the widespread use of M3N is the absence of evidence that this is a clinically useful tool in terms of improved outcome. In our experience the tool has been helpful in the planning and performing of surgery, although we acknowledge the possibility of observer bias. Epilepsy surgery does not easily lend itself to randomised controlled trials, and we would suggest uptake critical evaluation and reporting of these techniques and comparison with currently treated cohorts.

## Conclusion and further developments

We routinely use fully integrated multimodal images in the presurgical evaluation and surgical planning of epilepsy cases. Having shown the feasibility of the intraoperative M3N approach and its application to icEEG placement, we are now establishing a clinical trial to demonstrate utility and cost-effectiveness.

In addition, we are developing a new, semi-automated software programme to replace the Amira-based prototype, that will be quicker, easier to use and more practical in the environment of a busy epilepsy surgery centre. Despite the feasibility of routine intraoperative M3N as shown in this paper, the availability of dedicated software platform for M3N would facilitate more widespread uptake.

## Disclosure

John Duncan has received Institutional grant support from Eisai, UCB Pharma, GSK, Janssen Cilag, Medtronic, and GE Healthcare. Andrew McEvoy has received support from UCB, Baxter, and Cyberonics. The remaining authors have no conflicts of interest. We confirm that we have read the Journal's position on issues involved in ethical publication and affirm that this report is consistent with those guidelines.

## Figures and Tables

**Table 1:** The typical geometric parameters of the typical images utilised in M3N.

Image type		Matrix size	Voxel size (mm)
Navigation image (with MR-contrast fiducials): gadolinium enhanced T1 MPR		512 × 512 × 144	0.5 × 0.5 × 1.5
Diagnostic anatomical images	T1 FSPGR	256 × 256 × 166	0.94 × 0.94 × 1.1
	T2 FLAIR	256 × 160 × 32	0.94 × 1.5 × 3.5
Vascular images	MRV Time of Flight	256 × 256 × 93	0.94 × 0.94 × 2
	3D Phase Contrast MR	256 × 256 × 160	0.85 × 0.85 × 1
Functional imaging (fMRI): T2* GE planar (acquired 96 × 96 inplane)		128 × 128 × 58	1.87 × 1.87 × 2.5
White matter fibre tracking - Diffusion Tensor Imaging (DTI): T2 SE planar (acquired 96 × 96 inplane)		128 × 128 × 60	1.87 × 1.87 × 2.4
Ictal perfusion: SPECT		128 × 128 × 49	3.9 × 3.9 × 3.9
Metabolism: FDG PET		128 × 128 × 47	1.95 × 1.95 × 3.3
Localisation of icEEG electrodes: CT		512 × 512 × 172	0.4 × 0.4 × 1
