# Supplementary material for: Feasibility of multimodal 3D neuroimaging to guide implantation of intracranial EEG electrodes
Source: Epilepsy Res. 2013 Nov;107(1-2):91–100. doi: 10.1016/j.eplepsyres.2013.08.002 (PMC3830177; doi:10.1016/j.eplepsyres.2013.08.002)
Supplement: Supplementary file 1 [file mmc1.doc]

# Supplementary material 1

Table 1. Patient information.

| **Patient no** | **Presumed seizure onset zone prior to implantation** | **Implanted EEG electrodes** | **Lesion** | **3D models available pre-operatively and (intra-operatively). Intraop is a subset of preop** | **Benefits from use of multimodality 3D models** |
| --- | --- | --- | --- | --- | --- |
| 1 | Lt FL, pre-central gyrus | 8x8 grid Lt FL; 2x8 grid Lt PL; two 4-contact depths anterior and posterior to lesion; two 6-contact strips medial Lt FL | Pre-central cavernous angioma or DNT | (Lesion, iSPECT, foot&hand motor fMRI) | Targeting lesion; confirmation of central sulcus and motor cortex to plan craniotomy and place the 8x8 grid |
| 2 | Lt FL, SFG perilesional | 8x8 grid Lt FL; 2x8 grid Lt PL; two 1x4 depth electrodes ant and through lesion | FCD in Lt SFG | Language fMRI, iSPECT, CST, (lesion, hand motor fMRI, veins, cortex) | Confirmation of central sulcus and lesion; planning craniotomy and electrode placement |
| 3 | Lt OL, perilesional | Three 6-contact depths to the lesion and ant/post to it. Two 6-contact depths into amygdala and ant hippocampus | T2 FLAIR signal change on upper bank of calcarine fissure junction Lt OL | (Lesion, cortex, OR tract, veins) | Avoiding damage of optic radiation tract and veins; targeting lesion |
| 4 | Ant inf Lt FL, perilesional | Three 6-contact depths in ant Lt FL; three 6 contact depths in amygdala, hippocampus and Lt TL pole | DNET close to rectus gyrus in Lt FL | Lesion, (cortex, veins) | Avoiding damage of veins, electrode placement in relation to lesion |
| 5 | Rt FL | 8x8 grid post Rt FL and Rt STG; two 4x4 high density grids medial frontal lobe; two 6-contact strips SFG and medial PL | 2 non-specific calcifications in interhemispheric fissure close to foot motor area | CST, (Veins, iSPECT, Lt hand motor fMRI) | Localisation of presumed seizure onset zone (iSPECT) for complete coverage by electrodes; confirmation of central sulcus |
| 6 | Lt medial SFG | 8x8 grid Lt FL and PL; 4x8 high density grid interhemispheric medial FL; high density 2x8 grid interhemispheric medial foot motor cortex; 4-contact depth Lt medial PL | N/A | (R hand and foot motor fMRI, two MEG dipoles, veins, cortex) | Avoiding damage to veins; confirmation of location of CS |
| 7 | Rt fronto-parietal | 6x8 grid Rt PL and posterior FL; 4x8 high density Rt interhemiric medial PL; 2x8 high density Rt SMA; 2 strips Rt FL; depth into lesion | FCD in Rt sup parietal lobule adjacent to postcentral gyrus | (cortex, veins, lesion) | Planning craniotomy; targeting lesion |
| 8 | Lt temp pole; Lt OF; Lt medial FL | Lt hemisphere depth electrodes: 1x8 OF; 1x6 TL pole; 1x6 amygdala; 1x6 ITG; 1x6 post hippocampus; 1x6 STG | N/A | (cortex, veins, EEG-fMRI, PET) | Avoiding damage to veins; optimal coverage of possible seizure onset zone (EEG-fMRI) |
| 9 | Lt FL (premotor/OF cortex) | 6x8 grid Lt FL and PL covering motor area; 1 strip ant FL; 2x8 grid inf FL; three 1x6 depth electrodes into post, middle and ant SMA; 1x4 depth into lesion | FCD; Lt post IFG | (cortex, CST, lesion, lang fMRI) | Confirmation of CS (motor fMRI and cortex); planning craniotomy, placement of electrodes |
| 10 | Lt FL/TL | 4x8 grid Lt lateral TL; 4x8 high density grid Lt inf TL; 1x6 strip L OF; 1x6 strip Lt TL/OL; 1x6 depth Lt amygdala; 1x6 depth L hippocampus | N/A | (MEG dipole, veins, cortex) | Targeting irritative zone (MEG of IED) as possible seizure onset zone; avoiding damage to veins when placing depth electrodes; optimal coverage of Lt TL |
| 11 | Rt TL | R hemisphere depth electrodes: four 1x6 into Rt amygdala, ant&post hippocampus, temp pole; 1x8 into Rt OF region | N/A | (cortex, veins) | Avoiding damage to veins |
| 12 | Lt medial FL, SSMA/premotor | 8x8 grid Lt FL/PL; two 1x6 strips Lt FL pole; 1x6 strip Lt ant medial FL; 4x4 high density grid Lt SMA; 4x4 high density grid Lt medial covering foot motor area | N/A | (cortex, veins, MEG dipole) | Optimal coverage of the presumed seizure onset zone (MEG of IED); planning craniotomy |
| 13 | Rt PL, Rt medial post TL | 1x6 depth electrodes Rt hemisphere: amygdala, hippocampus, medial OL/TL, posterior to lesion, anterior to lesion, into lesion, foot sensory area | Ischaemic lesion; Rt precuneus and Rt medial temporal adjacent structures | OR tract, (Lesion, Lt hand motor fMRI, veins, simulated depth electrodes) | Avoiding damage to veins and optic radiation; optimal targeting of the extensive lesion and eloquent cortex (hand motor) using depth electrodes only |
| 14 | Rt OF or TL pole | Three 1x8 depth electrodes in Rt hemisphere: medial OF, lateral OF, ant OF; four 1x6 depth electrodes in Rt TL: TL pole, amygdala, hippocampus, basal TL | N/A | (MEG dipole, veins, simulated depth electrodes) | Avoiding damage to veins; optimal coverage of presumed seizure onset zone (MEG of IED) |
| 15 | Lt TL/PL/OL junction | 4x8 grid Lt lat TL; 1x6 strip Lt post TL; 2x8 high density grid Lt inf TL; 4x8 high density grid Lt inf post TL; three 1x6 depth electrodes L TL: amygdala, ant & post hippocampus | FCD in Lt inf temp and fusiform gyrus | (cortex, veins, lesion) | Planning craniotomy |

Abbreviations: SFG/STG – superior frontal/temporal gyrus; ant – anterior; post – posterior; inf – inferior; OR – optic radiation; PL/FL/OL/TL – parietal/frontal/occipital/temporal lobe; FCD – focal cortical dysplasia; IED – interictal discharges; CS – central sulcus; IFG/ITG – inferior frontal/temporal gyrus; CST – cortico-spinal tract; DNET - dysembryoplastic neuroepithelial tumour; MEG-magnetoencephalogram; SSMA – supplementary sensorymotor area; SMA – supplementary motor area; iSPECT – ictal single photon emission computer tomography; PET – positron emission tomography.
