# Supplementary material for: Feasibility of multimodal 3D neuroimaging to guide implantation of intracranial EEG electrodes
Source: Epilepsy Res. 2013 Nov;107(1-2):91–100. doi: 10.1016/j.eplepsyres.2013.08.002 (PMC3830177; doi:10.1016/j.eplepsyres.2013.08.002)
Supplement: Supplementary file 2 [file mmc2.doc]

# Supplementary material 2

# Multimodal 3D neuroimaging pipeline for support of surgical pathway of epilepsy patients

### 1st clinical decision making point: Discussion of implantation strategy

All relevant test results are integrated in one anatomical space. The task is performed by an expert in neuroimaging.

**Input data**:

- anatomical imaging (shows details of anatomy without need for processing and analysis):
  - T1 FSPGR - space defining image (format: DICOM);
  - Images showing abnormalities further outlined for 3D display (e.g. T2 FLAIR; format: DICOM);
  - Images showing cortical surface veins: Time-Of-Flight (TOF) or 3D Phase Contrast (format: DICOM).
- functional imaging:
  - MEG. The dipoles localising source of interictal epileptic activity are further converted into 3D models. Commercial software Curry (Compumedics USA, www.neuroscan.com) is used to analyse MEG data to obtain the result. (format: Analyse)
  - Ictal SPECT. An image showing ictal cerebral perfusion is provided along with the corresponding space defining T1 image. (format: Analyse)
  - FDG PET. An image showing glucose metabolism is provided along with the corresponding space defining CT image. (format: DICOM)
  - DTI. An image showing a white matter tract (e.g. optic radiation tract) is provided along the corresponding space defining T1 FSPGR image. (format: Analyse)
  - FMRI. An image representing a Statistical Parametric Map showing task-related BOLD signal increase is provided along with the corresponding space defining Echo Planar image (either a mean/averaged EPI or a single EPI image). (format: Analyse). Note: processing of fMRI landmarks in the M3N pipeline does not depend on the experimental paradigm.
- Other imaging
  - Brain structure mask. It is a result of segmentation of a T1 image using Freesurfer (<https://surfer.nmr.mgh.harvard.edu/>) software. The image represents a set of masks encoding different brain areas using different numbers. (format: Nifti)

**Requirements to the input data:**

Towards specification of borders of brain areas revealed using brain mapping neuroimaging techniques: The maps of various characteristics of eloquent and epileptogenic cortex (e.g. BOLD activations, probabilistic white matter tracts) do not have clear borders. However, only models with clearly defined borders can be used on the commonly used neuronavigation systems (Medtronic and BrainLab). The borders of landmarks for the mapping images have to be specified by the experts in every technique of cortical mapping (M/ESI, fMRI, DTI, SPECT, PET, etc).

**Use of Amira**:

1. All available images are rigid-body (6 parameters) co-registered to T1 image (diagnostic image) which defines basis space for multimodal image reconstruction. Manual co-registration is used when necessary. Option of applying parameters of transformation is used when an image carrying results of processing (fMRI de/activations on EPI images, white matter tracts on DTI, areas of hypometabolism on PET images, etc) is supplied along with a co-registered space defining image. Example: (1) mean EPI image is coregistered to T1; (2) the resulting parameters of transformation are applied to the corresponding Statistical Parametric Map (SPM) image obtained using SPM package ([www.fil.ion.ucl.ac.uk/spm/](http://www.fil.ion.ucl.ac.uk/spm/)).
2. 3D model of blood vessels. Veins generate high signal on the TOF image. They are masked from TOF image using an “Arithmetic” tool. 3D representation of blood vessels located outside the brain (e.g. in the scalp) are removed: (1) the cortical mask is dilated and interpolated to reach skull (using Segmentation Editor); (2) this mask is applied to TOF image; (3) remaining vessels (due to imperfection of the mask) are removed manually using 3D Surface Editor.
3. 3D model of cortical surface is created based on the multilabel image representing whole brain segmentation performed using Freesurfer software. The volume rendered image of the brain is created by applying the cortical mask to the clinical T1 image and using Voltex tool to visualise the resulting image.
4. Manual segmentation (Segmentation Editor) is used when a landmark can be outlined only by an expert in a situation when methodology for automated delineation is not readily available. Examples: 3D models of lesions and abnormalities as on anatomical images; hypometabolism foci on PET images.
5. Visualisation (major ways):
   1. Projections on cutting planes: axial, coronal, sagittal, oblique with user-defined angle.
   2. 3D surface rendering.
   3. 3D volume rendering.
6. The resulting clusters showing different landmarks (lesion, language FMRI, motor/sensory FMRI, white matter tracts, EEG-fMRI, MEG, ictal SPECT, FDG-PET) are color-coded according to fixed palette.

### 2nd clinical decision making point: planning placement of intracranial electrodes

Result: 3D objects simulating intracranial electrodes are placed according to the optimal hypothesis about epileptogenic brain area. The task is performed by neurosurgeon and neurophysiologist.

**Use of Amira**:

1. Simulation of depth electrodes: 3D objects representing straight tubes of small radius connecting the target point in the brain and the entry point on the brain surface, that is then extrapolated to the scalp.
2. Grid electrodes are simulated by a surface object wrapped around a set of base points set on the cortical surface within the brain area that needs to be sampled by intracranial EEG. The base points are placed inside the square area of the size equal to the size of the grid electrode chosen for implantation.

### 3rd clinical decision making point: using the results of image fusion to plan implantation on the Stealth

Result: a navigational T1 image with fused into it masks for landmarks which have to be reproduced as 3D models on the neuronavigation station for intra-operative use. The task is performed by an expert in neuroimaging.

Additional images:

- navigation T1 MPR with fiducials on the scalp surface (format: DICOM)

**Use of Amira**:

1. All images carrying landmarks chosen by the operating neurosurgeon to be uploaded on the neuronavigation system are co-registered and resliced to the navigation T1 image with fiducials (usage of upsampling and nonlinear interpolation are advisable to use during reslicing to minimise loss of information).
2. The landmarks are added to the navigation T1 in the form of 3D labels using expression A*abs((B>X)-1)+(B>X)*Y, where A – navigation T1 image, B – image carrying the landmark, X – threshold to segment the landmark, Y – code for the 3D label on the resulting image. Arithmetic tool is used for this task.
3. The final image containing all landmarks is exported in DICOM format to be uploaded on the neuronavigation system.

### 4th clinical decision point: using image fusion following implantation of the intracranial electrodes to plan resective surgery

Result: Image of the implanted intracranial electrodes is reconstructed in the base space of the clinical T1 image. The task is performed by an expert in neuroimaging.

Additional images:

- CT image (MR T1 image when available) with intracranial electrodes in situ. (format: DICOM)

**Use of Amira:**

1. CT image is coregistered (rigid body coregistration) to the base space defining clinical T1 image.
2. The intracranial electrodes generating high signal on the CT image are segmented by thresholding followed by manual removal of the residual noise (using Surface Editor).
3. Visualisation of the electrodes using surface rendering and colour-coding.

### 5th clinical decision point: post-resection follow-up

Result: The resected brain area is added to 3D dataset in the form of a 3D model. The task is performed by an expert in neuroimaging.

Additional images:

- T2 and/or T1 anatomical images showing the resected brain area.

**Use of Amira:**

1. The post-resection anatomical images are coregistered to the base space defining T1 image.
2. The resected brain area is manually segmented (using Segmentation Editor) by an expert in neuroimaging.
3. The resulting binary mask is visualised in the form of a surface- or volume-rendered object in the 3D dataset.
